# Clogging-free microfluidics for continuous size-based separation of microparticles

**DOI:** 10.1038/srep26531

**Published:** 2016-05-20

**Authors:** Yousang Yoon, Seonil Kim, Jusin Lee, Jaewoong Choi, Rae-Kwon Kim, Su-Jae Lee, Onejae Sul, Seung-Beck Lee

**Affiliations:** 1 Department of Electronic Engineering, Hanyang Universtiy, 222 Wangsimni-ro, Seongdong-gu, Seoul, 04763, Korea; 2 Department of Life Science and Research Institute for Natural Sciences, Hanyang Universtiy, 222 Wangsimni-ro, Seongdong-gu, Seoul, 04763, Korea; 3 Institute of Nano Science and Technology, Hanyang Universtiy, 222 Wangsimni-ro, Seongdong-gu, Seoul, 04763, Korea.

## Abstract

In microfluidic filtration systems, one of the leading obstacles to efficient, continuous operation is clogging of the filters. Here, we introduce a lateral flow microfluidic sieving (μ-sieving) technique to overcome clogging and to allow continuous operation of filter based microfluidic separation. A low frequency mechanical oscillation was added to the fluid flow, which made possible the release of aggregated unwanted polystyrene (PS) particles trapped between the larger target PS particles in the filter demonstrating continuous μ-sieving operation. We achieved collection of the target PS particles with 100% separation efficiency. Also, on average, more than 98% of the filtered target particles were retrieved after the filtration showing high retrieval rates. Since the oscillation was applied to the fluid but not to the microfluidic filter system, mechanical stresses to the system was minimized and no additional fabrication procedures were necessary. We also applied the μ-sieving technique to the separation of cancer cells (MDA-MB-231) from whole blood and showed that the fluidic oscillations prevented the filters from being blocked by the filtered cancer cells allowing continuous microfluidic separation with high efficiency.

Separation of micro-scale particles has been an important issue in industrial, biochemical and clinical applications for identification and analysis of specific particles^1^,^2^,^3^. To achieve the goal, microfluidics has been actively adopted because it is able to manipulate micro-particles precisely. Microfluidic techniques for separating micro-particles can be divided into two categories: active and passive methods. Active methods use external forces such as electric field, magnetic field, acoustic wave and optical interaction, utilizing the particle’s specific dielectric, magnetic or optical properties^4^,^5^,^6^,^7^,^8^,^9^. Although these techniques produce systems with high separation efficiency and selectivity, they generally lack high throughput due to weak target interaction that limits specimen flow rate, or they require target labeling which are time consuming and may reduce specimen viability. Passive methods utilize distinctive physical characteristics of the particles such as, size, density, and, especially for cells, deformability^10^,^11^,^12^,^13^,^14^,^15^,^16^,^17^,^18^,^19^,^20^. These methods have simple design, relatively simple fabrication, and high throughput. However, they usually suffer from lower selectivity compared to the active methods.

In biomedical analysis, separation of living cells is required as a powerful diagnostic and prognostic tool. Active methods based on antibodies could be a good choice because they show high efficiency in selective separation and detection of target cells. However with limited processing speed and cell viability, they are generally inappropriate for clinical applications that require high throughput and intact cells. Passive separation techniques utilizing the cell size can be a better choice for cell separation^21^,^22^,^23^. For example, separating circulating tumor cells (CTCs) that propagate through the peripheral circulatory system are known to be larger and more rigid than hemocytes, which enables size and deformability based separation^18^,^21^,^22^,^24^,^25^.

A typical approach among passive separation methods is to use microfluidic filters containing micro-fabricated pillars of specific interval or a sieve having micro-pores^10^,^12^,^13^,^18^,^20^. The microfluidic filters let particles with diameter smaller than the open pores pass through, while trapping targeted larger particles. However, as the trapped target particles accumulate, parts of the filters become blocked, reducing the number of open pores and they change the hydrodynamic resistance unpredictably. This results in aggregation of the smaller non-target particles around the constricted pores leading to contamination of the filtered target particles and lowering their purity^15^,^18^,^26^. It also induces higher shear stress, resulting in the trapped particles squeezing through the filters, or in the case of cells, the shear may induce rupture lowering the efficiency. Furthermore, hydraulic pressure applied to the filtered particles causes them to be immobile. Especially for live biological cells including non-targeted ones, pressurized contact to the filter surface gives high chances for non-specific adhesion^27^. This immobilization of targets hinders downstream analysis where retrieval of filtered particles is required. Even though the microfluidic filtration devices are simple in working principle and easy to fabricate, such critical issues have kept them from being widely utilized. To avoid these issues, systems incorporating actuated membranes or alternate fluid flow have been developed to prevent device clogging^28^,^29^,^30^,^31^,^32^,^33^,^34^. Those systems showed improved selection efficiency, but they required constant monitoring for the transition between static phase and dynamic phase, which limits processing speed and automation.

Here, we report on a lateral flow microfluidic sieving (μ-sieving) device that has overcome the limitation by adopting sieving which can be observed in our daily lives: for example, separation of grains and gravels from sand using a sieve. By adding a low frequency oscillation to the fluid flow, it was possible to purify the trapped target particles from smaller non-target particles. With the oscillated flow, the device successfully filtered polystyrene (PS) particles continuously without any contamination by smaller non-target particles. We also applied the device to the separation of a breast cancer cell line (MDA-MB-231) from whole blood without filter clogging. The sieved particles (or cells) could also be retrieved after filtration providing the potential for downstream analysis and further post-processing. Our μ-sieving device achieved high specificity and throughput. Furthermore, as we demonstrated the separation of cancer cells from blood, μ-sieving may be applied in future liquid biopsy.

## Concept of **μ**-sieving

**Figure 1:**
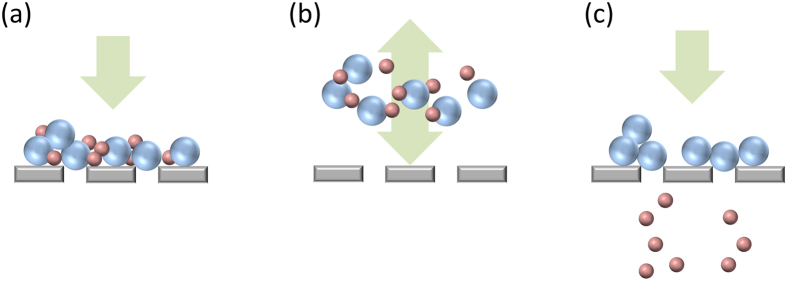
Schematic illustrations of the μ-sieving principle. (**a**) Clogged filter during steady flow filtration of particles. (**b)** In-phase vibration of filtered particles with fluid oscillation. (**c**) Passing of the smaller non-target particles through the filter.

To realize the action of sieving within a microfluidic filter environment, we applied oscillatory vibrations to the fluid, rather than to the filter as compared to previous reports^28^,^30^,^32^. This allowed the microfluidic chip and its filter to remain standing still, reducing mechanical stresses between different components in the microfluidic system, and keeping stable separation environment. Without fluid oscillation, the microfluidic filters cannot sustain extended operation due to eventual clogging (Fig. 1a). When the fluid oscillates, all the particles in the fluid normally oscillate collectively in phase, because the small variation in their microscopic size does not create enough of a difference in their inertial moment (Fig. 1b). However, when the oscillating particles come in contact with a filter, larger particles are trapped while smaller particles pass through (Fig. 1c).

## System Design and Simulation

**Figure 2:**
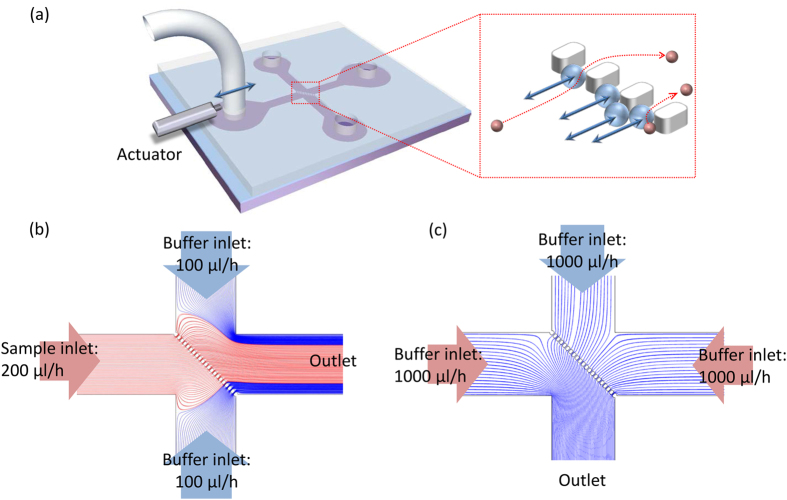
Illustration of the μ-sieving system. (**a**) Microfluidic chip with four channels and a piezoelectric actuator attached to the inlet tube. Red box shows the magnified view of the filter area. (**b**) Streamlines in the device during filtration and (**c**) retrieval of sieved particles. Red and blue indicate the sample and the buffer, respectively.

Figure 2 shows the schematic illustrations of the μ-sieving system. The figure visualizes the μ-sieving process with laminar flows. As shown in Fig. 2a, the device has an intersection of fluidic channels with a line of micro-pillars aligned diagonally at the intersection, in order to sieve particles longitudinally (Fig. 2b) and to retrieve transversely (Fig. 2c). To sieve micro-particles, the sample solution was injected from the left channel and the buffer solution was injected from the upper and lower channels, leaving the right channel as an outlet (Fig. 2b). For μ-sieving, a piezoelectric actuator was attached to the connection tube of the left channel inducing oscillation which caused the sample solution to move back and forth while advancing to the outlet. To retrieve the filtered particles, the buffer solution was injected from the upper, left, and right channel, leading the particles to the lower channel (Fig. 2c).

The fluid flow of the designed microfluidic geometry was analyzed using COMSOL multiphysics 5.1 to study the hydrodynamic conditions for μ-sieving and retrieving the target particles or cells. The simulation was performed under laminar condition, and the flow condition was controlled to properly trap and retrieve the target particles within our device geometry. The flow rate for the sample was set to be 200 μl/h, while sheath flows from the top and the bottom were 100 μl/h, respectively. For the retrieving mode, the bottom channel became the outlet, and the flow rates for the other three were set to be 1000 μl/h. With the streamline analysis, we were able to observe that the sample did not drain to the bottom channel, and the retrieving flow did not drain to the left channel as intended (Fig. 2b,c).

**Figure 3:**
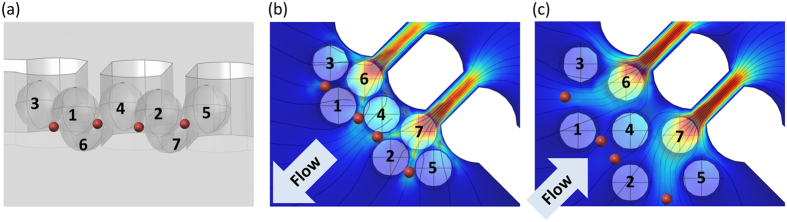
Illustrations of the μ-sieving analysis. (**a**) Simulation geometry showing the clogged filters. Red dots show the possible positions of the smaller particles. (**b**) Velocity field and streamlines at the time of transitioning to the backward oscillation and (**c**) the forward oscillation.

We also analyzed the principle of μ-sieving; how the smaller particles trapped around the larger particles could be released. We assumed that the larger particles (numbered particles in Fig. 3a) were stacked densely in front of the pillars during the forward flow, and the gaps between the stacked particles are small enough to block the passing of the smaller particles (red particles in Fig. 3a). During the backward flow by the oscillation, the stacked particles would recede with the fluid starting with the outermost particle (Fig. 3b). When the flow switches to the forward flow again, both of the smaller and larger particles start flowing at the same speed. However, as the larger particles start to impact on the filter, the gaps between the larger particles becomes narrower while the flow speed between them increases. Although the smaller particles were initially immobilized between the larger particles, now they will be swept along with the high speed inter-particle fluid, consequently escaping the stacks of larger particles and the filter (Fig. 3c).

## Device Fabrication

**Figure 4:**
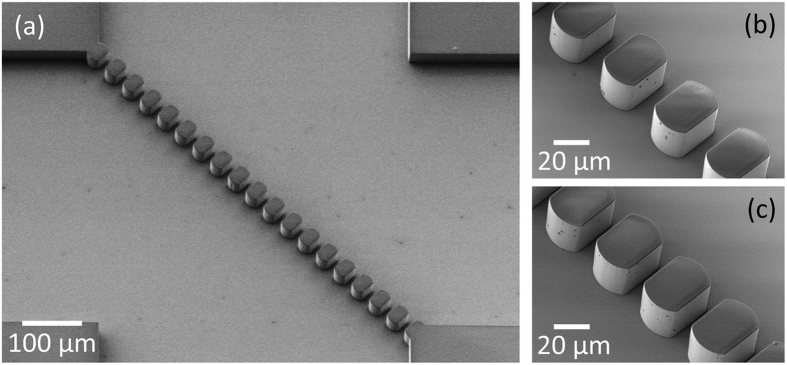
Scanning electron microscope images of the fabricated μ-sieve. (**a**) Diagonally aligned SU-8 (30 μm thick) micro-pillars acting as the filters. Interval of micro-pillars were (**b**) 12 μm for sieving PS particles, and (**c**) 7 μm for sieving cancer cells from whole blood.

The microfluidic device was realized on 2 × 2 cm^2^ surface oxidized silicon chips. Surpass 3000 (Dischem), an adhesion promoter, was spin coated on the SiO_2_ to enhance the adhesion of photoresist during the following processes. Photolithography was used to pattern the microfluidic channel in the SU-8 (2050, Microchem) photoresist which was spin-coated to be 30 μm thick. For encapsulation, 5 mm thick poly(dimethylsiloxane) (PDMS) was used with inlets and outlets defined. To have the device endure high fluidic stress, patterned SU-8 was exposed to O_2_ plasma (30 W, 160 mTorr, 30 s) followed by dipping in 5% (3-Aminopropyl)triethoxysilane for 10 minutes at 80 °C, then it was attached with the PDMS that was treated with O_2_ plasma (30 W, 160 mTorr, 30 s) resulting in firm Si-O-Si covalent bonds^35^. The width of the horizontal and vertical channels was 500 μm, and 35 × 25 μm^2^ round-edged pillars with specific intervals were aligned at 45° to the channel walls (Fig. 4a). The fabricated micro-pillars had intervals of 12 μm for the polymer microsphere sieving microfluidic chip (Fig. 4b), and 7 μm for cancer cell sieving from whole blood (Fig. 4c).

## Results and Discussion

### Characteristics of particle oscillation

**Figure 5:**
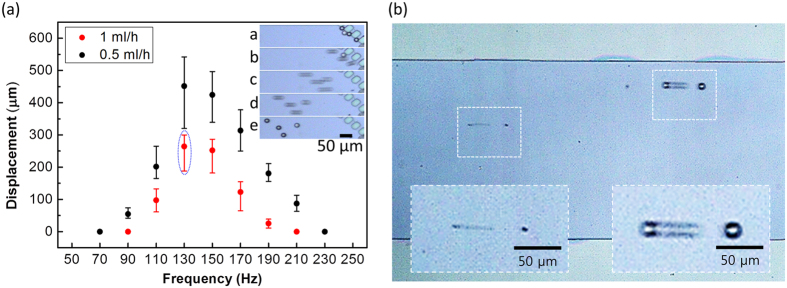
Characteristics of particle oscillation. (**a**) Measured particle displacement with varying actuation frequency and fluid flow rate. Inset figures (a–e) show sequential images of the μ-sieving at the time interval of 1 ms. (**b**) Trajectories of a small and a large PS particle. Inset figures are enlarged images of each particle oscillation.

We evaluated the movement of different sized particles within the oscillating fluid through a high speed camera. Figure 5a shows the oscillation amplitude of a 20 μm diameter PS microsphere observed for 0.5 ml/h and 1 ml/h flow speeds with oscillation frequencies between 70 Hz to 230 Hz, in 20 Hz steps. The oscillation amplitude was measured from the moment when the PS particles were blocked by the filter, to its maximum receding displacement position. The inset images in Fig. 5a shows the optical images of the filtered PS particles during the receding phase of its oscillation, from a to e in sequence, with the time interval of 1 ms. It can be seen that for both of the fluid flow speeds the maximum particle oscillation length was at 130 Hz. We believe that this frequency is near the resonant frequency of the total fluid mass contained within the microfluidic channel and the connecting tubes. This should vary with the microfluidic channel dimensions, the lengths of the inlet and outlet tubes connected with it, and the distance from the channel inlet to the piezoelectric actuation along the inlet tube. The dependence of the oscillation amplitudes on the fluid speeds was a result of the difference in the average forward motion of the particles during the receding phase of their oscillation. We then examined the oscillation according to the size of the particles, and discovered that the particle displacement was exactly the same regardless of their sizes for the same flow speed (Fig. 5b). Judging from their low particle Reynolds number (<0.1), this is due to the small sizes of the microspheres not creating enough of a difference in their relative movements^14^.

### Demonstration of μ-sieving and retrieval of PS micro-particles

**Figure 6:**
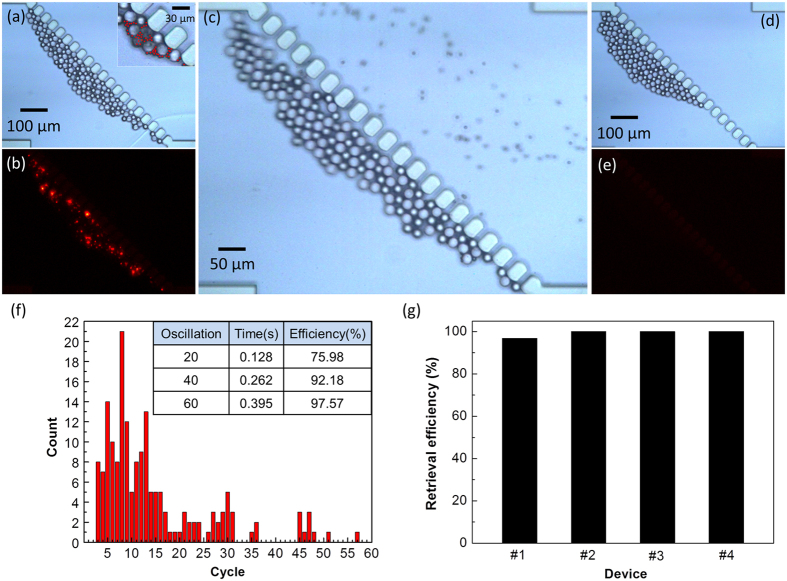
Demonstration of μ-sieving and retrieval of PS micro-particles. (**a**) Bright field and (**b**) fluorescent images before the activation of sieving. Red dots indicate the smaller non-target particles (5 μm) which were surrounded by the larger particles (20 μm). (**c**) Image of the smaller particles being released with the activation of fluid oscillation. (**d**) Bright field and (**e**) fluorescent images after fluid oscillation. (**f** ) The recorded number of smaller particles released during μ-sieving depending on the oscillation cycle. Inset table shows the time duration and the particle release efficiency (measured as the percentage of the number of smaller particles released) up to the given oscillation cycle. (**g**) Larger particle retrieval efficiency of the μ-sieving systems. The average value was 99.2%.

To quantitatively analyze the μ-sieving technique and test the feasibility of μ-sieving CTCs from whole blood, PS microspheres of 20 μm and 5 μm were utilized representing the size of CTCs and red blood cells (RBCs), respectively. Initially, the μ-sieving chip was operated without fluid oscillation resulting in the smaller PS particles aggregated among the larger PS particles filtered by the micro-pillars (Fig. 6a). Figure 6b shows the fluorescent image of smaller PS particles in Fig. 6a. Immediately after operating the piezoelectric actuator, the longitudinal oscillation started to release the small PS particles, allowing them to pass through the micro-pillar openings (Fig. 6c, see Supplementary Video S1). After only a second of oscillation, it can be seen in Fig. 6d and 6e that all of the small PS particles were released. Upon detailed evaluation (Fig. 6f), we find that most of the particles were released within the first 20 cycles of oscillations (0.128 s), and the rest of the particles were released gradually afterwards by advancing forward in the filter with each oscillation cycle. After 60 cycles (0.395 s), we observed that more than 97% of the trapped particles were released. Finally, the last two particles were released after 0.8 s of oscillation. The μ-sieving with fluid oscillation did not allow any larger PS particles to pass through the filter, showing 100% separation efficiency. The filtered larger PS particles were then retrieved by changing the flow direction. The retrieval experiment was repeated for 4 μ-sieving chips, and the average retrieval efficiency was 99.2% (Fig. 6g). Three of the four experiments showed 100% retrieval of the filtered PS particles. There were four larger PS particles trapped between the micro-pillars for device #1 which may be due to the slightly wider micro-pillar openings. It is generally reported that in micro-filter or membrane based microfluidic separation devices, retrieval rate of filtered particles have not been satisfactory due to some particles being squeezed into the filter openings. This is from the hydraulic pressure gradually increasing as the number of open filters becomes reduced by clogging. In our μ-sieving device, clogging was absent due to the fluidic oscillation; target particles were released from the filter repeatedly by backward flow, and so they did not experience such pressure between the pillars. This resulted in higher release efficiency.

### Cancer cell μ-sieving from whole blood

As proof-of-concept of cancer cell separation, we applied the μ-sieving technique to the separation of fixed cancer cells from whole blood. As with the previous experiments, fixed MDA-MB-231 cells were initially trapped prior to whole blood injection (see Supplementary Figure S2a). To reduce the hydraulic pressure on the filtered tumor cells, the sample flow rate was reduced to 0.2 ml/h, and the buffer flow rate was reduced to 0.1 ml/h. In normal flow condition, the shear rate at the filters increases with the trapping of cancer cells due to the reduction of fluid cross-section inducing platelet activation and raising thrombogenicity of whole blood^36^,^37^,^38^. The coagulation of the hemocytes with the cancer cells led to complete blocking of the filters preventing further operation. In most filter based whole blood CTC separation systems, to avoid this type of coagulation, lysis of RBCs, centrifuge separation of RBCs and the anti coagulant, or both was performed prior to the experiments^7^,^13^,^33^. However, this process may lead to the loss of CTCs which may critically hinder its application due to the relevant number of CTCs being very small (few out of 10^9^ hemocytes)^39^. Here, we avoided most of the pretreatments to remove the RBCs to demonstrate that it was possible to filter cancer cells directly from whole blood with minimum chemical treatment. We observed that the coagulated cancer cells were freed from the filter and started oscillating with the fluid when the fluid oscillation (130 Hz) was applied. This enabled the RBCs to pass through the filters allowing continued operation of the μ-sieve (see Supplementary Figure S2b and Supplementary Video S3).

**Figure 7:**
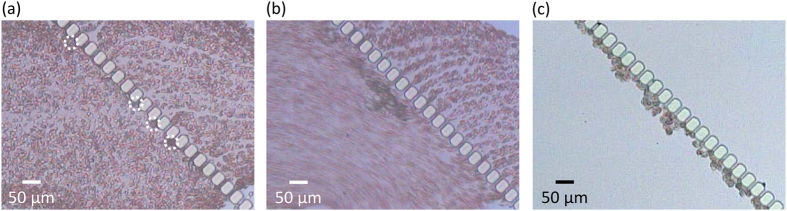
Optical microscope images of continuous μ-sieving of cancer cell spiked blood. (**a**) The filters partially blocked by captured cancer cells prior to fluid oscillation. White dotted circles indicate the cancer cells. (**b**) Continuous μ-sieving with filter pores unblocked by fluid oscillation. (**c**) The filtered cancer cells after completing μ-sieving with continuous fluid oscillation.

**Figure 8:**
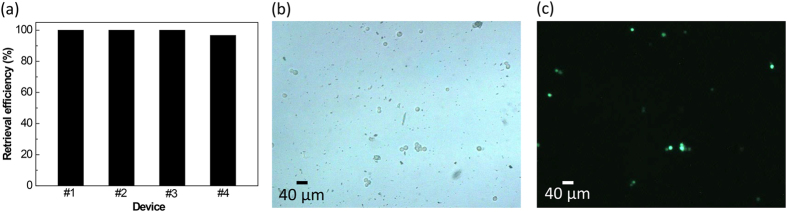
Retrieval of μ-sieved cancer cells. (**a**) Retrieval efficiencies measured from four μ-sieving chips (average retrieval efficiency of 99.2%). (**b**) Optical microscope image of retrieved cancer cells observed under bright field and (**c**) fluorescence.

Figure 7 shows the result of continuous μ-sieving applied to cancer cell spiked blood. We began the experiment without fluid oscillation which led to several cancer cells being captured by the filters. It can be seen that the fluid flow through the filter pore with cancer cells was reduced significantly increasing the flow through the rest of the unblocked pores (Fig. 7a). If the high shear rate continues, it may lead to coagulation, making the filtered cancer cells irretrievable. However, as fluid oscillation was applied at an early stage of the experiment, we see that the filtered cancer cells began to oscillate with the fluid freeing the filter pores (Fig. 7b, see Supplementary Video S4). This allowed the whole blood specimen (0.2 ml) to be filtered continuously for one hour, and resulted in the filtration of 49 cancer cells (Fig. 7c). No cells conforming to the size and morphology of the fixed cancer cells was observed in the outlet resulting in complete separation. By reversing the fluid flow as shown in Fig. 2c, it was possible to collect the filtered cancer cells with the average retrieval efficiency of 99.2% (Fig. 8a). Optical microscope image of the retrieved particles shows many different types, ranging from circular cells to sharp debris (Fig. 8b). From the microscope image taken under fluorescence, most of the circular cells with proper morphology were identified as green fluorescent protein (GFP) overexpressed cancer cells that were spiked into the whole blood (Fig. 8c). Other smaller particles visible in Fig. 8b may be hemocytes that were not completely drained flowing back through the filter during flow reversal for particle retrieval, which does lower purity. Sufficient rinsing after sieving should reduce reverse flow contamination and improve purity.

The simplicity of the method of μ-sieving and its effectiveness is noteworthy. By simply contacting a piezoelectric actuator with the inlet tube, existing filtration systems can also have greatly improved selectivity without structural modification, freedom from clogging by small particle impurities, increased throughput and retrieval efficiency. Such improvements achieved by μ-sieving will lead to effective post-processing and downstream analysis. However, further research is required to utilize μ-sieving in clinical application. The key issue for live cell manipulation is in their deformability making size-based separation more difficult. Viability of the cells exposed to oscillation is also an important issue that should be addressed.

## Conclusion

We have developed a μ-sieving system which is capable of size-based separation of PS particles and cancer cells. Different from conventional filtration systems, we added fluid oscillations that enabled continuous operation without filter clogging. The fluid oscillation was induced by a piezoelectric actuator making contact with the inlet tube. The maximum amplitude of particle oscillation was at the actuation frequency of 130 Hz. PS particles of 20 μm were successfully filtered from 5 μm PS particles showing 100% separation efficiency and purity. Sieved particles were retrievable with 99.2% efficiency by changing the flow direction. We also demonstrated μ-sieving of MDA-MB-231 spiked whole blood, and showed continuous sieving of cancer cells without blood coagulation and cell loss. With the ability to allow continuous operation of size-based filtered separation, our device may bring closer application of microfluidic separation devices to biochemical analyte separation, clinical liquid biopsy and industrial micro-particle separation.

## Materials

PS microspheres were utilized to characterize the μ-sieving. Two different diameters of PS, 20 μm (Sigma-Aldrich) and 5 μm (Micromod), were chosen. The smaller PS particles have red fluorescence when exposed to UV radiation for effective visualization of the particle separation. The PS microspheres were suspended in a 20% sucrose solution to expand the time of sinking during the experiments, and surfactant, 0.1% F-127 (Sigma-Aldrich), was also included to disperse the microspheres preventing aggregation.

For μ-sieving cancer cells, MDA-MB-231, a human breast cancer cell line, spiked blood was utilized. Written informed consent was obtained from healthy donors at Hanyang University Seoul Hospital with an approval granted by the Hanyang University Institutional Review Board. All methods were carried out in accordance with the approved guidelines. The blood was diluted twice with 1× phosphate buffer saline. MDA-MB-231 was established from the American Type Culture Collection (Manassas, VA). The cells were tagged by GFP and grown in Dulbeco’s modified eagle’s medium supplemented with 10% fetal bovine serum, penicillin (100 units/ml), streptomycin (100 g/ml) and cultured in a humidified 5% CO_2_ atmosphere at 37 °C. Cultured tumor cells were harvested and fixed in 70% ethanol prior to the experiments.

## Additional Information

**How to cite this article**: Yoon, Y. *et al*. Clogging-free microfluidics for continuous size-based separation of microparticles. *Sci. Rep.* **6**, 26531; doi: 10.1038/srep26531 (2016).

## Supplementary Material

Supplementary Information

Supplementary Video S1

Supplementary Video S3

Supplementary Video S4

Supplementary Video S6
