# Supplementary material for: Clogging-free microfluidics for continuous size-based separation of microparticles
Source: Sci Rep. 2016 May 20;6:26531. doi: 10.1038/srep26531 (PMC4873827; doi:10.1038/srep26531)
Supplement: Supplementary Information [file srep26531-s1.pdf]

Clogging-free microfluidics for continuous size-based separation of microparticles

Yousang Yoon[a], Seonil Kim[a], Jusin Lee[a], Jaewoong Choi[a], Rae-Kwon Kim[b], Su-Jae Lee[b], Onejae Sul[c], and Seung-Beck Lee[a,c*]

[a]Department of Electronic Engineering, Hanyang Universtiy, 222 Wangsimni-ro, Seongdong-gu, Seoul, 04763, Korea

[b]Department of Life Science and Research Institute for Natural Sciences, Hanyang Universtiy

[c]Institute of Nano Science and Technology, Hanyang Universtiy

***corresponding author**

(Phone: 82-2-2220-1676; Fax: 82-2-2294-1676; E-mail: sbl22@hanyang.ac.kr)

Supplementary Video S1. Demonstration of μ-sieving (PS particles). The smaller PS particles that were initially trapped and immobilized among the larger particles are observed to be released with fluid oscillation and no longer being trapped.

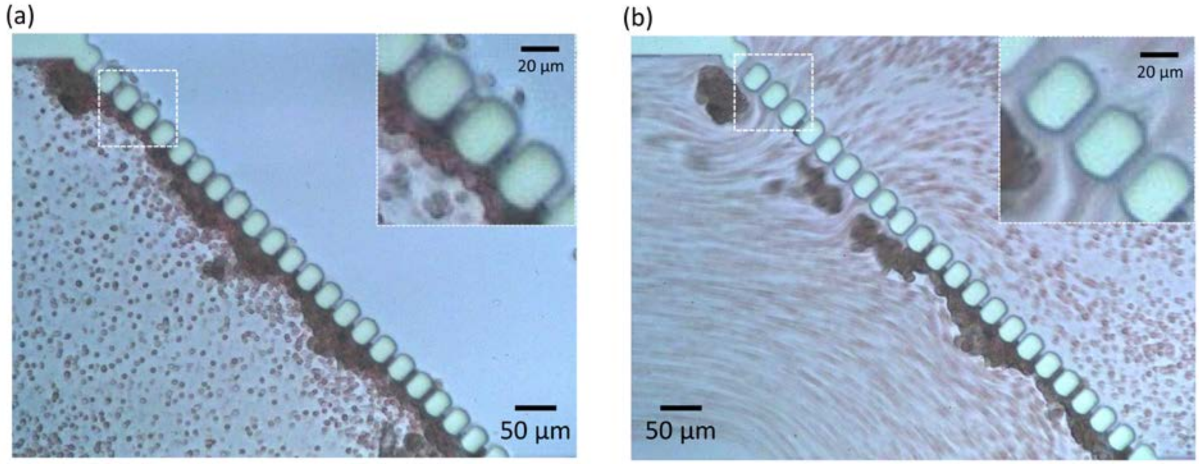

Supplementary Figure S2. Optical microscope images of cancer cell μ-sieving from whole blood. (a) Filters fully clogged before μ-sieving. (b) RBCs released during activation of fluid oscillation.

Supplementary Video S3. Demonstration of μ-sieving (MDA-MB-231 spiked blood). Unblocking of fully clogged filters can be observed with fluid oscillation starting from the weakly coagulated filters. Since the initiation of fluid oscillation, further coagulation cannot be observed.

Supplementary Video S4. Demonstration of continuous μ-sieving (MDA-MB-231 spiked blood). Fluid oscillation was applied after several cancer cells were captured.

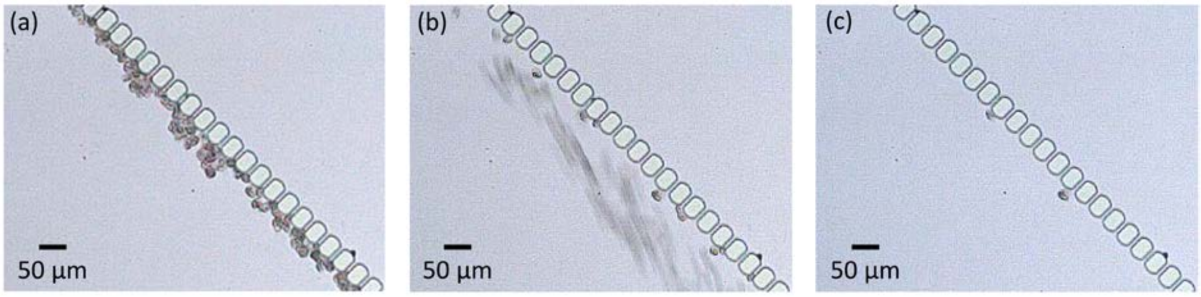

Supplementary Figure S5. Optical microscope images of cancer cell retrieval. (a) Filtered cancer cells prior to retrieval, (b) during retrieval by reverse flow, and (c) after retrieval. Two cancer cells were observed to remain attached to the filters. We assume that this may be due to the time elapsed between specimen preparation and the end of the filtration process being too long (over 5 h) causing the activation of fibrin from the whole blood to attach to the trapped cells.

Supplementary Video S6. Demonstration of cancer cell retrieval. The video shows retrieval of the filtered cancer cells as seen in Fig. 7c and Supplementary Fig. S5a.
